# Serum insulin-like growth factor-1 and its binding protein 3 as prognostic factors for the incidence, progression, and outcome of hepatocellular carcinoma: a systematic review and meta-analysis

**DOI:** 10.18632/oncotarget.19186

**Published:** 2017-07-12

**Authors:** Jing Wang, Yu-Chuan Li, Min Deng, Hai-Yin Jiang, Li-Hua Guo, Wen-Juan Zhou, Bing Ruan

**Affiliations:** ^1^ Department of Cardiology, The First Affiliated Hospital, School of Medicine, Zhejiang University, Hangzhou, Zhejiang, China; ^2^ State Key Laboratory for Diagnosis and Treatment of Infectious Diseases, Collaborative Innovation Center for Diagnosis and Treatment of Infectious Diseases, The First Affiliated Hospital, School of Medicine, Zhejiang University, Hangzhou, Zhejiang, China; ^3^ Department of Health Management Center, Wuxi Third People's Hospital, Wuxi, Jiangsu, China

**Keywords:** insulin-like growth factor-1, IGF-binding protein-3, hepatocellular carcinomas, overall survival, time-to-progression

## Abstract

**Purpose:**

Previous studies have supported an association between serum insulin-like growth factor-1 (IGF1) and IGF-binding protein 3 (IGFBP3) levels and hepatocellular carcinoma (HCC), but the results were inaccurate. It has recently been proposed that IGF1 and IGFBP3 play roles in the time-to-progression (TTP) and overall survival (OS) of HCC patients. Our results revealed that serum IGF1 level is predictive of the progression and survival of HCC patients.

**Results:**

HCC was associated with a significant reduction in serum IGF-1 and IGFBP-3 levels compared to cirrhosis (*p* = 0.037). Low serum IGF1 levels were predictive of a shorter TTP (OR, 2.74; 95% confidence interval [CI], 1.92–3.90) and poorer OS (odds ratio [OR], 2.20; 95% CI, 1.81–2.68) in HCC patients. The IGF1/IGFBP3 molar ratio was not significantly associated with the risk of HCC (OR, 1.311; 95% CI, 0.761–2.260).

**Materials and Methods:**

We conducted a comprehensive literature search in PubMed, EMBASE, and the Cochrane Library. Twenty studies met the inclusion criteria and were subjected to statistical analysis. The geometric mean and standard deviation (SD) of serum IGF1 and IGFBP3 levels in the healthy, cirrhosis, and HCC groups were calculated. Pooled odds ratios (ORs) were calculated using a fixed-effects model to analyse the association of serum IGF1 level with the progression and survival of HCC patients.

**Conclusions:**

Serum IGF1 and IGFBP3 levels were positively associated with the incidence of HCC. Serum IGF1 level is an independent prognostic factor for the progression and survival of HCC patients.

## INTRODUCTION

Hepatocellular carcinoma (HCC) is the third leading cause of cancer-related deaths worldwide. In the past two decades, the role of the IGF axis in the pathogenesis of various neoplasms, including HCC, has been a focus of research [1]. Some case–control studies have supported a positive association between the insulin-like growth factor-1 (IGFI) level and the risk of liver cancer [2–4]. Indeed, in clinical practice, IGFI is used to assess HCC reserve capacity [5, 6] and as a prognostic marker for HCC progression and survival [3]. However, further research is needed.

Insulin-like growth factor-1 (IGF1), which is synthesised by the liver, is an important regulator of cellular proliferation, differentiation and apoptosis [7–9]. These effects can be inhibited by IGFBP3, which binds to and prevents IGFI from binding to type 1 insulin-like growth factor receptor (IGFI-R) [10]. About 99% of circulating IGF1 is bound to IGF-binding proteins, with most bound to IGF-binding protein 3 (IGFBP3). Less than 1% of circulating IGF1 is in an unbound form [11]. The IGF system is involved in the pathogenesis of several malignancies, including breast, prostate, colorectal, and gastric cancer [7, 9]. The development of HCC is reportedly correlated significantly with low IGFI and IGFBP3 levels and a high IGFI/IGFBP3 molar ratio [12–14]. Additionally, low baseline serum IGF1 levels are reportedly associated with shorter time-to-progression (TTP) and poorer overall survival (OS) in patients with HCC, irrespective of the grade of hepatic dysfunction [15, 16]. However, these studies were hampered by their low quality and small sample sizes. We performed a systematic review and meta-analysis of 20 studies to investigate the associations of IGF1 components with HCC and to examine their ability to predict the survival and prognosis of HCC patients.

## RESULTS

### Literature search and selection

**Figure 1:**
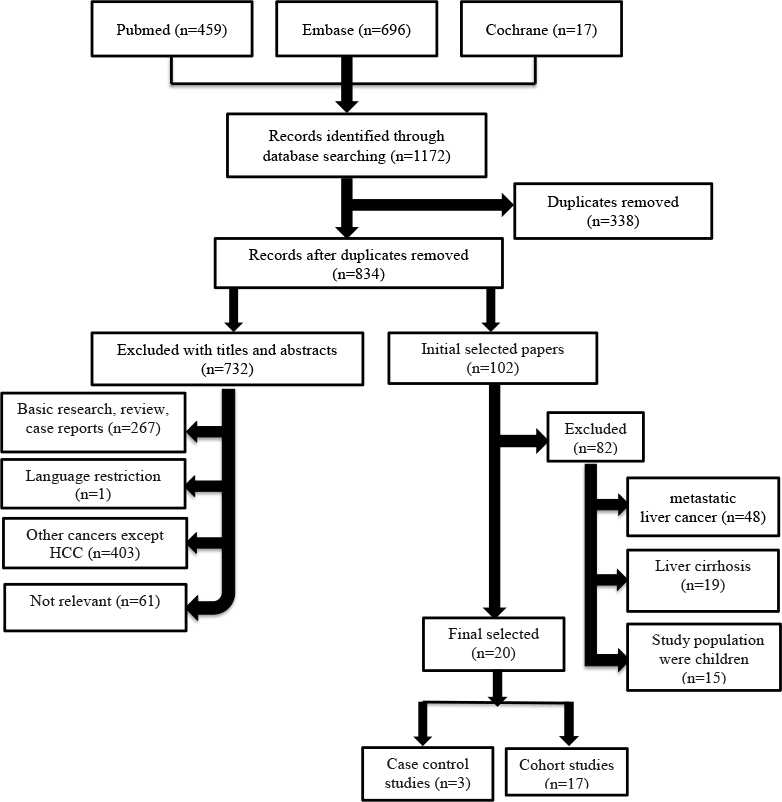
Flow chart of the studies selected for systematic review and meta-analysis

Initially, 1172 records were retrieved by searching the Cochrane Library, PubMed, and EMBASE databases. After removal of duplicates, 834 articles were screened. Of these articles, 732 were excluded by reading the titles and abstracts, and the remaining 102 articles underwent detailed evaluation of the full text. A further 82 studies were excluded as they did not meet the inclusion criteria. Finally, 20 studies were included in our systematic review and meta-analysis: 3 case–control and 17 cohort studies (Figure 1). Eleven studies investigated the roles of IGF-1, IGFBP3, and the IGF-I/IGFBP3 molar ratio in the development of HCC (Table 1A), and nine studies assessed the ability of serum IGF1 level to predict the TTP and OS of HCC patients (Table 1B).

Table 1Characteristics of studies included in the review

**Table:** (A) Studies of serum IGF-1 and IGFBP-3 levels and IGF-I/IGFBP-3 molar ratio among healthy, cirrhosis and HCC groups

Study, year	Location, setting	Study design	Study period	Age, y	IGF-1/IGFBP-3 assay	HCC diagnosis	HCC, *n*	Cirrhosis case, *n*	Healthy controls, *n*	Adjusted confounders
Stuver et al, 2000^2^	USA, Hospital-based	Case control	1995–1998	39–88	Chemiluminescence assay	Biopsy, AFP, US	73	NV	111	Gender, age
Mazziottiet et al, 2002^14^	Italy, Hospital-based	Cohort study	1995–2000	43–81	immunoradiometric assay	US, biopsy	20	84	NV	Gender, age
Mattera et al, 2003^12^	Italy, Hospital-based	Cohort study	NA	25–75	immunoradiometric assay	US, Biopsy	63	40	150	Age, diet
Cujic et al, 2010^39^	Serbia, Hospital-based	Cohort study	2008	20–75	radioimmunoassay	US, CT	8	24	31	N A
Major et al, 2010^25^	USA, population-based	Cohort study	1985–1988	50–69	ELISA	AFP, US, CT, MRI, biopsy	50	NA	400	Age,
Su et al, 2010^22^	Taiwan, Hospital-based	Cohort study	2005–2006	20–83	radioimmunoassay	Biopsy, AFP, US	65	NA	165	Recruitment center, date of blood collection
Rehem et a,l 2011^40^	Egypt, Hospital-based	Cohort study	2011	24–69	Immunoradiometric assay	CT or/and AFP	20	60	20	NA
Aleem et al, 2012^41^	Egypt, Hospital-based	Cohort study	2010–2011	NA	ELISA	US, AFP	62	79	100	Age, height, BMI
Adamek et al, 2013^42^	Poland, Hospital-based	Cohort study	2010–2012	18–63	immunoenzymetric assay, ELISA	USAFP	61	37	15	Age, gender
Lukanova et al, 2014^3^	European countries, population-based	nested case-control study	2002–2006	35–75	ELISA	AFP, US, CT, MRI, biopsy	125	NA	247	Recruitment center, gender, age, date of blood collection
Adachi et al, 2016^4^	Japan, population-based	nested case-control study	1997–2000	40–79	immunoradiometric assay	AFP, US, CT, MRI, biopsy	91	NA	263	gender, age, and residential area, Hepatitis viral infection, body mass index, smoking, and alcohol intake

**Table:** (B) Ability of serum IGF1 level to predict the progression and survival of HCC patients

Study, year	Location, setting	Study design	Follow-up Period, m	age(mean or median),y	IGF-1/IGFBP-3 assay	HCC diagnosis	Cases, *n*	Outcomes index
Treiber et al 2006^26^	Germany, Hospital-based	Cohort study	6	65.7	ESILA	US, CT, MRI	71	OS, TTP
Kaseb et al, 2011^27^	USA, Hospital-based	Cohort study	96	60	ELISA	biopsy, US	288	OS
Shao et al, 2012^28^	Taiwan, Hospital-based	Cohort study	40	54	ELISA	biopsy, AFP	83	OS, PFS
Cho et al, 2013^13^	Korea, Hospital-based	Cohort study	72	57.1	immunoradiometric assay	AFP, US, CT, MRI, biopsy	91	OS, TTR
Cho et al, 2014^29^	Korea, Hospital-based	Cohort study	41.8	56	immunoradiometric assay	AFP, US, CT, MRI, biopsy	155	OS, TTP
Kaseb et al, 2014^6^	USA, Hospital-based	Cohort study	16.5	60	ELISA	AFP, US, CT, MRI, biopsy	155	OS
Abdel-Wahab et al, 2015^15^	Egypt, Hospital-based	Cohort study	6.5	63.2	ELISA	Biopsy, CT	100	OS
Elmashad et al, 2015^16^	Egypt, Hospital-based	Cohort study	8	51	ELISA	CT or /and AFP	89	OS, TTP
Liu, 2016^36^	China, Hospital-based	Cohort study	47	55	ELISA	AFP, US, CT, MRI, biopsy	128	OS, TTP

### Characteristics of participants

The characteristics of the participants are presented in Table 1A and 1B. The study involved 1,798 HCC patients, 324 cirrhosis patients, and 1,502 healthy controls. The studies were published from 2000 to 2016. Seventeen were hospital-based studies, and three were population-based studies. Six studies were performed in Europe, four in the United States, four in Africa, and six in Asia. Of the 20 studies, 3 were case–control studies, and 17 were cohort studies; all involved only adults. Serum IGF1 and IGFBP3 levels were tested by enzyme-linked immunosorbent assay (ELISA) in all of the studies. The diagnosis of HCC was established based on biopsy, elevated alpha-fetoprotein level (AFP), and imaging techniques—such as ultrasound (US), three-phase dynamic computed tomography (CT), and magnetic resonance imaging (MRI)—according to the guidelines of the American Association for the Study of Liver Diseases [17].

### Serum IGF1 and IGFBP-3 levels in the healthy, cirrhosis, and HCC groups

The geometric mean serum levels of IGF1 and IGFBP3 differed significantly among the healthy, cirrhosis, and HCC groups independently of the degree of impairment of liver function (Table 2). The serum IGF-I and IGFBP3 levels of the healthy group were significantly higher than those of the cirrhosis group (*p* < 0.01). HCC was associated with lower serum IGF1 and IGFBP3 levels compared to liver cirrhosis (*p* = 0.037). The effects of different detection methods (ELISA and radioimmunoassay), research designs (case–control, cohort, and cross-sectional study), settings (hospital- and population-based), and locations (Asia, North America, and Europe) on serum IGF1 levels were assessed. In HCC patients, there was no difference between cohort and cross-sectional studies (*p* = 0.056) or between data collected in hospital- and population-based settings (*p* = 0.367) (Table 3). Additionally, we assessed the association between the IGFI/ IGFBP3 molar ratio and the risk of HCC, odds ratio (OR, 1.311; 95% confidence interval (CI), 0.761–2.260; I-square, 46.86; *p* = 0.130) not showing a significant increase of IGF-I/IGFBP3 ratio in HCC incidence (data not shown).

**Table 2:** Serum IGF1 and IGFBP-3 levels of the healthy, cirrhosis, and HCC groups

	*n*	Mean ± SD	*p* value
**Serum IFG-1 level (ng/ml)**			
Healthy population	1255	181.34 ± 107.89	< 0.001
Cirrhosis patients	324	117.77 ± 109.69	0.037
HCC patients	432	102.91 ± 85.89	
**Serum IFGBP3 level (ng/ml)**			
Healthy population	665	2608.35 ± 787.27	< 0.001
Cirrhosis patients	156	1278.84 ± 777.30	0.026
HCC patients	175	1092.38 ± 736.06	

**Table 3:** Factors associated with serum IGF1 levels in the healthy, cirrhosis, and HCC groups

	Health control			Cirrhosis			HCC		
Factors and subset	*n*	Mean ± SD	*p* value	*n*	Mean ± SD	*p* value	*n*	Mean ± SD	*p* value
**Detection methods**									
ELISA	626	190.61 ± 104.78	0.002	116	190.69 ± 139.42	< 0.001	165	118.71 ± 93.41	0.005
Radioimmunoassay	629	172.10 ± 110.20		208	77.11 ± 57.73		176	90.68 ± 90.48	
**Research designs**									
Case-control study	374	129.14 ± 63.20	< 0.001				53	65.87 ± 45.99	0.008
cohort study	415	145.69 ± 50.26	< 0.001	121	68.00 ± 33.50	< 0.001	70	91.94 ± 57.63	0.056
cross-section study	466	254.98 ± 131.05		203	147.44 ± 127.29		218	117.52 ± 106.54	
**Location settings**									
hospital-based	592	236.75 ± 124.85	< 0.001	324	117.77 ± 109.69		291	102.36 ± 98.12	0.367
population-based	663	131.86 ± 54.19					50	115.2 ± 51.61	
**Study locations**									
Asia	548	209.26 ± 143.03	< 0.001	139	168.28 ± 139.32		147	144.19 ± 114.71	< 0.001
North America	511	152.47 ± 54.12	< 0.001				103	89.82 ± 54.51	< 0.001
Europe	196	178.53 ± 73.61		185	79.82 ± 56.46	< 0.001	91	56.04 ± 51.40	

### Association of IGF1 level with TTP and OS

**Figure 2:**
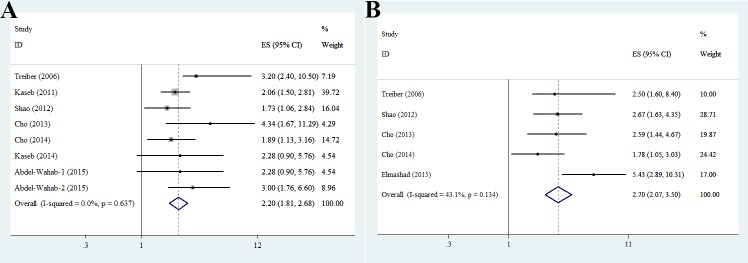
Association between serum IGF1 level and HCC (**A**) Overall survival (OS); (**B**) time-to-progression (TTP).

Low IGF1 levels were predictive of a shorter TTP (OR, 2.74; 95% CI, 1.92–3.90) and poorer OS (OR, 2.20; 95% CI, 1.81–2.68) in HCC patients, irrespective of the grade of hepatic dysfunction. No significant heterogeneity was observed among the studies. This suggests serum IGF1 level to be an independent prognostic factor for the survival (Figure 2A) and progression (Figure 2B) of HCC patients. The subgroup analysis indicated that use of different detection methods (ELISA and radioimmunoassay), research designs (clinical and cohort studies), and locations (Asia, North, America and Europe) had no significant influence on the TTP and OS of HCC patients (Table 4A and 4B).

Table 4Subgroup analysis of the association between serum IGF1 level and HCC

**Table:** (A) Overall survival (OS)

Factors	Studies, *n*	OS for HCC (95%CI)	*I*^2^	Model used
**Detection method**				
ELISA	6	2.311 (1.858—2.874)	31.4%	Fixed effects
Radioimmunoassay	2	2.279 (1.448—3.587)	55.6%	Fixed effects
**Study design**				
Clinical trail	3	2.073 (1.615—2.660)	0%	Fixed effects
Cohort	5	2.745 (1.993—3.781)	31.8%	Fixed effects
**Study location**				
Asia	5	2.489 (1.922—3.223)	41.6%	Fixed effects
North America	2	2.082 (1.546—2.802)	0%	Fixed effects
Europe	1	3.2 (2.4—10.5)

**Table:** (B) Time-to-progression (TTP)

Factors	Studies,*n*	TTP for HCC (95%CI)	*I*^2^	Model used
**Detection method**				
ELISA	3	3.275 (2.305—4.653)	43.1%	Fixed effects
Radioimmunoassay	2	2.109 (1.422—3.128)	0%	Fixed effects
**Study design**				
Clinical trail	2	2.623 (1.721—3.997)	0%	Fixed effects
Cohort	3	2.873 (1.530—5.394)	71.4%	Random effects
**Study location**				
Asia	4	2.718 (2.062—3.583)	57.1%	Fixed effects
Europe	1	2.5 (1.6—8.4)		

### Serum IGF1 levels according to clinical characteristics

The associations between clinical factors and baseline IGF1 levels are shown in Table 5. HCC patients with the hepatitis C virus (HCV) infection had significantly lower IGF1 levels than those with the hepatitis B virus (HBV) infection (*p* = 0.034). HCC patients of Child–Pugh class C (*p* = 0.002), Barcelona Clinic Liver Cancer Stage-B (BCLC-B) (*p* < 0.01), or BCLC-C (*p* = 0.019) had significantly lower baseline IGF1 levels. However, baseline IGF1 levels did not differ significantly according to age, gender, serum AFP level, tumour nodularity, or vascular invasion (*p* > 0.05).

**Table 5:** Serum levels of IGF1 according to the clinical characteristics of HCC patients

Factor and subset	*n*	Mean ± SD	p value	Relative mean and SD
**Age (years)**				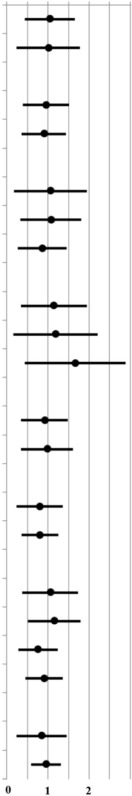
< 60	333	99.42 ± 58.50	0.584	
≥ 60	195	96.23 ± 73.59		
**Gender**				
Female	140	90.24 ± 53.50	0.4	
Male	323	85.76 ± 52.08		
**Hepatitis infection status**				
None	36	101.58 ± 84.73	0.962	
HBV	337	102.18 ± 70.57	0.034	
HCV	57	81.25 ± 56.69		
**Child-Pugh class**				
A	336	109.12 ± 76.76	0.653	
B	137	112.94 ± 98.14	0.002	
C	79	158.75 ± 117.3		
**Serum AFP level(ng/ml)**				
< 200	264	87.45 ± 54.46	0.353	
≥ 200	120	93.23 ± 60.58		
**Tumor nodularity**				
Monofocal disease	155	76.19 ± 54.02	0.98	
Multifocal	113	76.34 ± 42.77		
**BCLC stage**				
0	45	100.56 ± 64.74	0.376	
A	163	109.73 ± 60.53	< 0.001	
B	136	72.34 ± 46.13	0.019	
C	99	86.29 ± 43.05		
**Vascular invasion**				
No	147	80.21 ± 57.86	0.166	
Yes	70	90.62 ± 34.48		

### Publication bias

**Figure 3:**
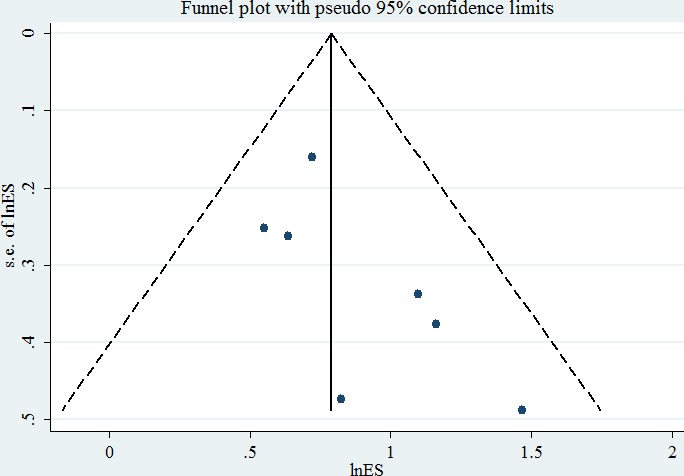
Publication bias in studies of the association between serum IGF1 level and overall survival of HCC patients

No evidence of publication bias was detected in nine studies of the ability of serum IGF1 level to predict the OS rate of HCC patients (Begg test, *p* = 0.083; Egger test, *p* = 0.103) (Figure 3).

## DISCUSSION

High levels of circulating IGF-I and low levels of IGFBP-3 are reportedly associated with an increased risk of several common cancers, including those of the prostate, breast, colon, and lung [18–20]. However, no systematic review has evaluated the associations of IGF1 and IGFBP3 levels with HCC. We conducted a systematic review and meta-analysis of the association of serum IGFI and IGFBP3 levels with the risk of HCC. Low levels of IGF1 and IGFBP-3 were positively associated with the risk of HCC independently of the degree of impairment of liver function, consistent with previous reports [2, 21, 22]. Additionally, patients with cirrhosis had lower circulating IGF1 and IGFBP3 levels than healthy controls (*p* < 0.001). This is likely because of reduced secretion of the liver-derived factors IGF1 and IGFBP3 in cirrhosis and HCC patients with chronic liver damage and functional insufficiency [23].

The free form of IGF1 induces cell proliferation and inhibits apoptosis in bone, cartilage, the central nervous system, and the kidneys. The IGF1 to IGFBP3 molar ratio represents the level of active IGF1 [24], and patients with an IGF1 concentration higher than their IGFBP3 level are at an increased risk of liver cancer [25]. However, no significant association between the IGFI/IGFBP3 molar ratio and the risk of HCC (OR, 1.311; 95% CI, 0.761–2.260) was detected.

In HCC patients, a low serum IGF1 level was predictive of a shorter TTP (OR, 2.74; 95% CI, 1.92–3.90) and poorer OS (OR, 2.20; 95% CI, 1.81–2.68), with no heterogeneity (I-square = 0), irrespective of the grade of hepatic dysfunction, which is consistent with previous reports [6, 13, 15, 26–29]. Nine of the studies used identical statistical methods: (1) receiver-operating characteristic (ROC) curves for censored survival data to identify the optimum cut-off value for predicting outcome; [30] and (2) Cox proportional hazard regression analysis to evaluate independent risk factors for disease progression and OS [31] after adjustment for age, gender, HBV/HCV infection, AFP level, Child–Pugh class, BCLC stage, tumour nodularity, and vascular invasion. Serum IGF1 level was the most significant predictor of HCC progression and survival. A prospective study reported that a low IGF-I concentration at least 5 years before cancer diagnosis was associated with an increased risk of liver cancer [25]. Further, larger-scale studies of the ability of ILGF-1 level to predict HCC are warranted.

Additionally, circulating IGF-1 level was correlated with virus infection, Child–Pugh class, and BCLC stage of HCC patients. Therefore, the circulating IGF-1 level was associated with HCC progression. The circulating IGF-1 level is reportedly significantly correlated with survival, the synthetic function of the liver, and tumour parameters [27, 32]. Moreover, integrating plasma insulin-like growth factor-1 level into the Child–Turcotte–Pugh score (IGF-CTP score) resulted in improved risk stratification of HCC patients [6, 15]. The IGF-CTP score should thus be validated in further studies.

We analysed all available prospective studies that adjusted for potential risk factors, such as liver function and the clinical characteristics of HCC patients. However, this study had several limitations. First, the meta-analysis was vulnerable to the bias in the original studies, and the IGF1 and IGFBP3 assays and study design were not standardised, which led to different IGF1 and IGFBP3 levels among the studies. Future prospective studies should use a uniform study design and identical assays. Second, previous studies showed that energy and protein intake were associated with IGF1 and IGFBP3 concentrations^33,34^, but we did not adjust for dietary parameters. Third, most of the included studies involved hospitalised patients, who may not be representative of the general population, likely leading to overestimation of the risk of HCC. Finally, the number of eligible studies was small, which may have influenced the accuracy of the results.

In conclusion, circulating IGF1 and IGFBP3 levels were positively associated with the incidence of HCC, and the IGF1 level emerged as an independent prognostic factor for the progression and survival of HCC patients. Therefore, further large-scale, well-designed studies that consider a larger number of confounding factors are warranted.

## MATERIALS AND METHODS

### Search strategy

Case–control and cohort studies were identified by searching PubMed, EMBASE, and the Cochrane Library using the following keywords: Insulin-like growth factor I, insulin-like growth factor binding protein 3, and hepatocellular carcinomas. Only English-language publications were included in the study. The databases were searched for articles published up to October 2016.

### Selection of studies and data extraction

we included observational studies that met all of the following inclusion criteria: (1) case–control or cohort design; (2) use of adult subjects; (3) inclusion of a healthy control group and HCC patients with cirrhosis; (4) measurement of serum levels of IGF1 and/or IGFBP3 and calculation of means ± SDs; (5) calculation of odds ratio (OR) or relative risk (RR) of IGF1 serum level for HCC progression and survival; and (6) written in the English language.

### Data extraction and quality assessment

Data extraction was conducted independently by J.W. and Y.-C.L., and discrepancies were resolved by D.M., H.-Y.J., L.-H.G., and W.-J.Z. before the final analysis. The following data were collected from each study: author, year of publication, study country, study setting, total number of subjects in each group, serological detection method for IGF1 and IGFBP3, basis for HCC diagnosis, and statistical adjustments made. The quality of the included studies was assessed using the Newcastle-Ottawa Scale (NOS) [35], which was developed to assess the quality of nonrandomised studies in meta-analyses. On this scale, observational studies are scored in three categories: selection (four questions) and comparability (two questions) of the study group, and ascertainment of the outcome of interest (three questions). All questions have a score of one, with the exception of those addressing the comparability of study groups, for which separate points are awarded for controlling for age and/or sex (maximum, two points). Studies with more than five points were included in the meta-analysis; one study [36] did not meet this criterion and was excluded from the analysis of the survival of HCC patients (Supplementary Figure 1).

### Outcomes

The primary analysis assessed the association of serum IGF1 and IGFBP3 levels with the incidence of HCC and the ability of a low IGF1 serum level to predict HCC progression and survival. The serum IGF1 levels of the HCC patients were analysed according to their clinical characteristics, such as age, gender, AFP level, Child–Pugh class, BCLC stage, tumour nodularity, and vascular invasion.

### Statistical analysis

statistical analysis was conducted using STATA 12.0 software (StataCorp LP, College Station, TX, USA). The Cochran Q chi-square test and the I^2^ statistic were used to assess heterogeneity among the studies [37]. An I^2^ value of > 50% or a *p*-value of < 0.05 for the Q-statistic was taken to indicate significant heterogeneity [38]. We used a fixed-effects model to estimate pooled odds ratios (ORs) and corresponding 95% confidence intervals (CIs) for HCC progression and survival. Additionally, the serum IGF1 and IGFBP levels of the healthy, cirrhosis, and HCC groups were subjected to one-way analysis of variance (ANOVA). Serum IGF1 levels were analysed according to the clinical characteristics of the HCC patients. Publication bias was analysed using a Begg funnel plot and the Egger test [43].

## SUPPLEMENTARY FIGURE


