# Supplementary material for: Serum insulin-like growth factor-1 and its binding protein 3 as prognostic factors for the incidence, progression, and outcome of hepatocellular carcinoma: a systematic review and meta-analysis
Source: Oncotarget. 2017 Jul 12;8(46):81098–108. doi: 10.18632/oncotarget.19186 (PMC5655265; doi:10.18632/oncotarget.19186)
Supplement: Supplementary file 1 [file oncotarget-08-81098-s001.pdf]

Serum insulin-like growth factor-1 and its binding protein 3 as prognostic factors for the incidence, progression, and outcome of hepatocellular carcinoma: a systematic review and meta-analysis

SUPPLEMENTARY MATERIALS

A

NOS for Assessment of Quality of Included Studies: Cohort Studies										
Quality assessment criteria	Acceptable*	Mazziotti et al, 2002	Major et al, 2010	Adamek et al, 2013	Cho et al, 2013	Cho et al, 2014	Kaseb et al, 2014	Abdel-Wahab et al, 2015	Elmashad et al, 2015	Liu et al, 2016
Selection										
Representativeness of exposed cohort?	Representative of average adult in community (age/sex/being at risk of disease)	*	*	*	*	*	*	*	*	—
Selection of the nonexposed cohort?	Dawn from same community as exposed cohort	*	—	*	*	*	*	*	*	*
Ascertainment of exposure?	Secured records, structured interview	*	*	*	*	*	*	*	*	*
Demonstration that outcome of interest was not present at the start of the study?	Only incident cases of HCC	*	*	*	—	—	—	—	—	—
Comparability										
Study controls for age/sex	Yes	*	*	*	*	*	*	*	*	*
Study controls for at least 3 additional factors	Yes	—	*	*	*	*	*	*	*	*
Exposure										
Assessment of outcome?	Independent blind assessment, record linkage	*	*	*	*	*	*	*	*	—
Was follow-up evaluation long enough for outcome to occur?	Yes	*	—	—	*	—	—	*	*	—
Adequacy of follow-up evaluation of cohorts?	Complete follow-up evaluation, or subjects lost to follow-up evaluation unlikely to introduce bias	*	*	—	—	—	*	*	*	—
Overall quality score (maximum=10)		8	7	7	7	6	7	8	8	4

B

NOS for Assessment of Quality of Included Studies: Case-Control Studies				
Quality assessment criteria	Acceptable*	Stuver et al, 2000	Lukanova et al, 2014	Adachi et al，2016
Selection				
Is the case definition adequate?	Yes, with independent validation	*	*	*
Representativeness of cases?	Consecutive or obviously representative series of cases	*	*	*
Selection of controls?	Community controls	—	*	*
Definition of controls?	No history of HCC	*	*	*
Comparability				
Study controls for age/sex	Yes	*	*	*
Study controls for at least 3 additional factors	Yes	—	—	*
Exposure				
Ascertainment of exposure?	Secure record, structured interview where blind to case-control status	*	*	*
Same method of ascertainment of cases/controls?	Yes	*	*	*
Nonresponse rate?	Same for both the group	—	—	—
Overall quality score (maximum=10)		6	7	8

Supplementary Figure 1: The newcastle-Ottawa quality assessment scale (NOS).
